# Effect of Roux-en-Y Gastric Bypass Surgery on Bile Acid Metabolism in Normal and Obese Diabetic Rats

**DOI:** 10.1371/journal.pone.0122273

**Published:** 2015-03-23

**Authors:** Hina Y Bhutta, Neetu Rajpal, Wendy White, Johannes M. Freudenberg, Yaping Liu, James Way, Deepak Rajpal, David C. Cooper, Andrew Young, Ali Tavakkoli, Lihong Chen

**Affiliations:** 1 Department of Surgery, Brigham and Women's Hospital, Boston, Massachusetts, United States of America; 2 Harvard Medical School, Boston, Massachusetts, United States of America; 3 Department of Investigative Medicine, Imperial College, London, United Kingdom; 4 Metabolic Drug Discovery, GlaxoSmithKline Inc., Research Triangle Park, North Carolina, United States of America; 5 Molecular Discovery Research, GlaxoSmithKline Inc., Research Triangle Park, North Carolina, United States of America; 6 Quantitative Sciences Division, GlaxoSmithKline Inc., Research Triangle Park, North Carolina, United States of America; INRA, FRANCE

## Abstract

In addition to classic functions of facilitating hepatobiliary secretion and intestinal absorption of lipophilic nutrients, bile acids (BA) are also endocrine factors and regulate glucose and lipid metabolism. Recent data indicate that antiobesity bariatric procedures e.g. Roux-en-Y gastric bypass surgery (RYGB), which also remit diabetes, increase plasma BAs in humans, leading to the hypothesis that BAs may play a role in diabetes resolution following surgery. To investigate the effect of RYGB on BA physiology and its relationship with glucose homeostasis, we undertook RYGB and SHAM surgery in Zucker diabetic fatty (ZDF) and normoglycemic Sprague Dawley (SD) rats and measured plasma and fecal BA levels, as well as plasma glucose, insulin, Glucagon like peptide 1 (GLP-1) and Peptide YY (PYY), 2 days before and 3, 7, 14 and 28 days after surgery. RYGB decreased body weight and increased plasma GLP-1 in both SD and ZDF rats while decreasing plasma insulin and glucose in ZDF rats starting from the first week. Compared to SHAM groups, both SD-RYGB and ZDF-RYGB groups started to have increases in plasma total BAs in the second week, which might not contribute to early post-surgery metabolic changes. While there was no significant difference in fecal BA excretion between SD-RYGB and SD-SHAM groups, the ZDF-RYGB group had a transient 4.2-fold increase (P<0.001) in 24-hour fecal BA excretion on post-operative day 3 compared to ZDF-SHAM, which paralleled a significant increase in plasma PYY. Ratios of plasma and fecal cholic acid/chenodeoxycholic acid derived BAs were decreased in RYGB groups. In addition, tissue mRNA expression analysis suggested early intestinal BA reabsorption and potentially reduced hepatic cholic acid production in RYGB groups. In summary, we present novel data on RYGB-mediated changes in BA metabolism to further understand the role of BAs in RYGB-induced metabolic effects in humans.

## Introduction

Obesity is fast becoming a worldwide epidemic and is driving an unprecedented surge in type 2 diabetes mellitus (T2DM). The World Health Organisation estimates that 500 million people worldwide will suffer from T2DM by 2050 and describes the rise of such chronic non-communicable diseases as an ‘impending disaster’ (http://www.who.int/mediacentre/news/releases/2011/ncds_20110427/en/). Rapid and sustained improvements in glucose homeostasis following bariatric operations have led to their classification as metabolic procedures and gold standard treatments for diabetic subjects with significant obesity (BMI>35kg/m^2^) [1,2]. The mechanisms of post-operative T2DM remission can be divided into weight loss-dependent and independent pathways. Although weight–loss dependent results are common to all bariatric operations, the Roux-en-Y gastric bypass (RYGB) procedure has demonstrated early and weight-independent improvements in T2DM [3,4]. The mechanisms underlying this observation are under intense investigation and incretin hormones, nutrient sensors/transporters, intestinal glucose utilization, and gut microbiota are all receiving attention for putative roles in mediating the weight loss-independent effects [5–9].

Consistent data from human and animal studies show that RYGB surgery significantly increases plasma bile acid (BA) levels [10–14]. BAs have pleiotropic effects beyond their established role as “digestive surfactants” that promote absorption of lipids [15]. They are endocrine factors that activate different receptors (e.g. farnesoid-X receptor (FXR) and TGR5) [16] and stimulate the release of metabolic hormones such as Peptide YY (PYY) and Glucagon-like peptide-1 (GLP-1), which in turn regulate glucose and lipid metabolism [17,18] and enhance energy expenditure in brown adipose tissue [19]. Hence it is plausible that increased plasma BA levels after surgery have a role in the early and weight-independent improvement in insulin sensitivity and glucose handling. Meanwhile, disrupting enterohepatic recirculation of BAs with bile acid sequestrants or inhibitors of apical sodium dependent bile acid transporter (ASBT, Slc10a2), which reduce plasma BAs and increase fecal BA excretion, also improves glucose metabolism in humans [20] and animal models [21–23], questioning the metabolic significance of BA changes after RYGB. To further investigate mechanisms of RYGB-mediated changes in circulating BAs and the role of BAs in the remission of T2DM after RYGB, we studied the effects of this surgery on BA metabolism in lean normoglycemic Sprague Dawley (SD) as well as obese Zucker Diabetic Fatty (ZDF) rats.

## Methods

### Animals

The Harvard Medical Area Standing Committee on Animals prospectively approved all studies. Nine-week-old Sprague Dawley (Harlan Laboratories, Frederick, MD) and obese (*fa*/*fa*) Zucker Diabetic Fatty rats (Charles River Laboratories, Raleigh, NC) were acclimatized to a constant temperature and humidity (22°C and 70% respectively) for 5 days, with a fixed 12:12 light: dark cycle (lights on at 7am). All animals were male. The initial average body weights for SD and ZDF rats were 285.6±2.4g and 314.3±4.6g, respectively. A formula diet (Purina 5008: Carbohydrate 56.5%, Protein 26.8%, Fat 16.7%) was provided *ad libitum* except for the five days following surgery (as described below). Animals were caged in pairs pre-operatively and individually after surgery.

### Animal model

**Fig 1:**
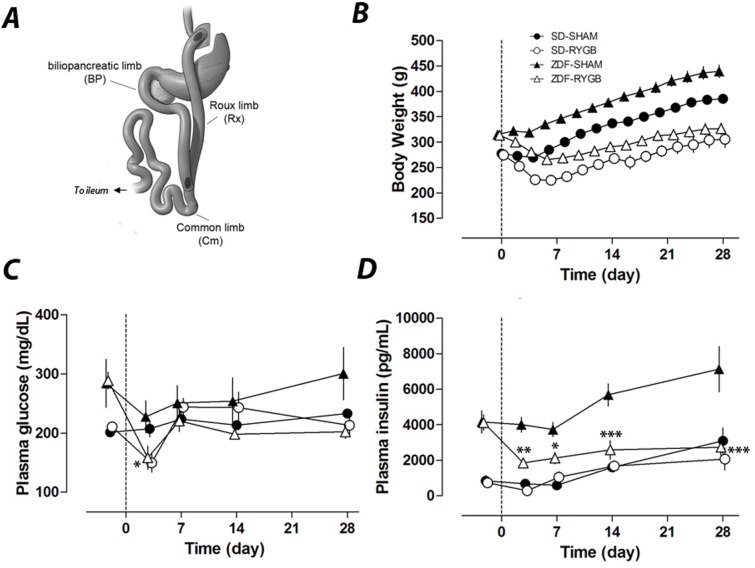
RYGB surgery and its effects on body weight, plasma glucose and insulin. RYGB and sham surgeries were performed in normal SD rats and diabetic ZDF rats on day 0. Body weight and plasma glucose and insulin were measured up to 28 days. ***A***. Schematics of RYGB surgery indicating the transactions and incisions intestine limbs. ***B***. Post-surgery body weights of RYGB groups were significantly different from those of corresponding sham groups (p<0.001) for all time points except day 3. Non-fasting plasma glucose (***C***) and plasma insulin (***D***) were measured on days −2, 3, 7, 14 and 28. * P<0.05, **P<0.01, ***P<0.001 vs. the corresponding sham group.

Animals were fasted from 7pm the evening prior to surgery, with *ad libitum* access to water. General anaesthesia was induced and maintained via inhaled isoflurane (2–3% in oxygen). Rats were administered 0.1mL Penicillin G (Phoenix Pharmaceuticals, Burlingame, CA) and RYGB was performed as described; through a midline laparotomy the stomach was divided using the Endo GIA Universal stapling device (2–2.5mm staples) (Covidien Medical Supplies, Mansfield, MA) just below the gastroesophageal junction from greater to lesser curve, taking care to preserve the vagus nerve in this region. The jejunum was divided 16cm distal to the Ligament of Treitz and the distal cut end anastomosed to a 1cm gastrotomy on the anterior surface of the gastric pouch (interrupted, 6/0 polydioxanone suture (PDS)). A 1cm enterotomy was made on the antimesenteric aspect of the jejunum 10cm distal to the gastrojejunostomy and anastomosed to the proximal cut end of jejunum as an end-to-side anastomosis (interrupted, 6/0 PDS), forming the biliopancreatic (BP) limb (illustrated in Fig. 1A). Sham rats underwent laparotomy with manual bowel manipulation for a 50 minute period to match the time taken to perform RYGB surgery. The abdomen was closed in 2 layers using 3/0 monocryl. Rats were kept on a heating pad throughout the operative period to minimize hypothermia and 10–15mL 0.9% Normal Saline was administered subcutaneously to maintain intra-operative hydration. 0.3% buprenorphine (Webster Veterinary, Fort Devens, MA) was administered as post-operative analgesia (0.05–0.1mL IM, bd) for a minimum of 48 hours. Post-operatively animals were fasted for 1 day with free access to tap water, before providing liquid diet *ad libitum* (CVS Liquid Nutrition 8oz: 250kcal, 40g carbohydrate, 9g protein, 6g fat). After 5 days animals were returned to pre-operative solid diet.

### Experiment Protocol

SD and ZDF rats were randomized to receive SHAM or RYGB surgery. Some RYGB animals died shortly after the surgery. Data from those animals that survived the 28 day study period are reported here (SD-RYGB, n = 6; SD-SHAM, n = 9; ZDF-RYGB, n = 11; ZDF-SHAM, n = 9). Stool and serum values were serially analysed until post-operative day (POD) 28 at which point the animals were sacrificed (under isoflurane anaesthesia) and tissue samples were collected. Body weights (commencing on the day of surgery) and food intake (once solid diet was resumed post-operatively) of all animals were documented on a daily basis. All blood samples were taken non-fasted at 9am 2 days pre-operation and on POD 3, 7, 14 and 28 under isoflurane anaesthesia for analysis of plasma biochemistry and hormones. Fecal pellets were collected over a 24 hour period on the same days. A wire rack was placed at the bottom of each cage throughout the 24 hour to prevent coprophagia, and all pellets were retrieved at the end of this time period. Fecal samples were kept at −80°C for subsequent analysis.

### Tissue harvest

All tissues were harvested at the same time (11am) to minimize variations due to circadian influences on gene expression. Under general anaesthesia as above, a midline laparotomy was performed and the following 1cm full-thickness segments of bowel were harvested from the mid-point of each limb unless otherwise stated (Fig. 1A): Roux limb (Rx), common limb (Cm), biliopancreatic (BP) limb, terminal ileum (TI, 1cm proximal to ileocecal valve), ascending colon (AC) and descending colon (DC). Finally a 1cm^3^ segment of the inferior right lobe of the liver was taken. Segments were immediately frozen in liquid nitrogen and stored at −80°C for subsequent RNA extraction. Orthotopically matched segments of bowel and liver were harvested from sham rats in the same manner.

### Plasma assays

Blood samples obtained at time points detailed above were added to chilled EDTA tubes containing Dipeptidyl Peptidase IV (DPPIV) inhibitor (10uL/mL blood; Millipore, Billerica, MA). After centrifugation at 4000 x g for 15 min at 4°C, plasma was removed and frozen at −80°C. Insulin, total PYY, and total Glucose-dependent insulinotropic peptide (GIP) were measured with the Milliplex MAP Rodent Gut Hormone Panel (Cat #: MGTMAG-78K), and active and total GLP-1 with the Meso Scale Discovery kits (Cat#: K150JWC and K150JVC respectively). Plasma glucose and total BAs were measured with the Olympus AU640 clinical chemistry analyzer (Beckman Coulter, Brea, CA).

### Fecal analysis

Fecal extraction was performed using a method described previously [21]. Fecal total BAs were measured with the Olympus AU640 clinical chemistry analyzer.

### RNA Preparation

RNA extraction was performed using the RNeasy Mini kit (Qiagen Inc, Valencia, CA). Total RNA yield and purity was estimated by UV spectroscopy (Nanodrop ND-1000 Spectrophotometer; Nanodrop Technologies, Wilmington, DE) and RNA quality was assessed on an Agilent 2100 Bioanalyzer (Agilent Technologies, Santa Clara, CA). Based on the quality of RNA extracted from all seven tissues, five animals were selected from each group for mRNA expression analysis.

### RT-PCR

First strand cDNA synthesis was performed using the Applied Biosystems High Capacity cDNA Reverse Transcription kit (Applied Biosystems, Foster City, CA) according to the manufacturer's instructions. Quantitative real-time PCR utilized custom made TaqMan® Low Density Array (TLDA) from Applied Biosystems (Foster City, CA) and followed the manufacturer's instructions. 50μL (12.5ng/μL) cDNA in 50μL of Applied Biosystems 2X Universal PCR Master mix was loaded onto each port of the TLDA plates. Thermal cycling was performed using an ABI Prism 7900HT Sequence Detection System. Preliminary data analysis was done using SDS v2.4 software (settings: manual baseline; threshold, 0.2; Life Technologies/Applied Biosystems, Grand Island, NY). The gene expression value of each gene was normalized to GAPDH expression.

### Bile acid species analysis

BA species were profiled using liquid chromatography-electrospray ionization-tandem mass spectrometry (LC-ESI-MS). Standards used were: CDCA, chenodeoxycholic acid; CA, cholic acid; αMCA, alpha-muricholic acid; βMCA, beta-muricholic acid; T-CDCA, taurochenodeoxycholic acid; G-CDCA, glycochenodeoxycholic acid; T-αMCA, tauro-alpha-muricholic acid; T-CA, taurocholic acid; G-CA, glycocholic acid; LCA, lithocholic acid; DCA, deoxycholic acid; UDCA, ursodeoxycholic acid; T-LCA, taurolithocholic acid; G-LCA, glycolithocholic acid; T-DCA, taurodeoxycholic acid; G-DCA, glycodeoxycholic acid; T-UDCA, tauroursodeoxycholic acid; G-UDCA, glycoursodeoxycholic acid.

### Statistical analyses

#### All data besides gene expression

All measurements other than the BA profiling results were analyzed as follows. Each response was fit to a linear mixed model with fixed effects for days, strains (SD/ZDF), treatments, and their interactions plus random effects for rats (to model subject-level variability). We computed the p-value for SHAM vs RYGB at each day for each rat type, and then we applied a multiplicity adjustment to reduce false positive findings. We applied the Benjamini-Hochberg false discovery rate (FDR) correction [24] across time points, separately for each type and response. Since rats were measured prior to treatment and at multiple days post-treatment, all results were adjusted for baseline. To adjust for baseline we used a model that assumed no surgical effect at Day = 0 (for body weight) and Day = −2 (for other responses). All figures display baseline-adjusted means plus or minus one standard error.

The BA profiling data were analyzed differently because many analytes were too low for detection (not detectable, denoted “ND”) by mass spectrometry, and because there were fewer time points but more analytes (approximately 20). Therefore, the exact Wilcoxon test was used to compare SHAM to RYGB for each rat type/source (plasma/feces)/day, because the Wilcoxon test properly accounts for the ND results. Then we applied the FDR multiplicity adjustment to the Wilcoxon p-values across analytes. The multiplicity adjustment was done separately for each type/source/day.

Surgical differences were considered statistically significant if FDR p-value ≤ 0.05. All analyses were done with SAS software (The SAS system for Windows. Release 9.2 TS2M3, SAS Institute, Cary, NC) or R software (R foundation for Statistical Computing, Vienna, Austria. URL http://www.R-project.org/). Graphs were prepared with R’s ggplot2 package (H. Wickham. ggplot2: elegant graphics for data analysis. Springer New York, 2009).

#### Gene expression data

Taqman expression values (i.e. 2^(40-Ct)^ value) were normalized by the corresponding average GAPDH expression value (i.e. 2^(40-Ct)^ value) taken over the corresponding technical replicates. Expression values where either technical replicate showed a Ct value greater than 36 were filtered out. Then, if fewer than 3 animals had data for a given gene and tissue type, all measurements for that gene in that tissue type were filtered out. Expression values were then log2 transformed for subsequent analyses. To determine the surgery effect, a linear model was defined with treatment (RYGB or sham surgery) and tissue type as experimental factors and also accounting for potential statistical dependency between samples due to the fact that for each animal, multiple samples (one per tissue type) were derived, as well as for the technical replicates. Model estimates were computed using the limma R package which implements an empirical Bayes method to robustly estimate variance after a linear model fit [25] followed by p-value adjustment to compute the FDR [24]. Genes were defined as differentially expressed if they were 1.5-fold up- or down-regulated and had an FDR ≤0.1.

## Results

### Body weight and food intake

Sham rats steadily and continuously gained weight post-operatively, reaching 140% of baseline weight by 28 days after surgery in both the SD and ZDF groups. In contrast, the RYGB groups lost weight, reaching nadirs of 81% (SD) and 85% (ZDF) of baseline by POD6 before increasing to 111% (SD-RYGB) and 103% (ZDF-RYGB) of baseline by POD28 (Fig. 1B).

Mean daily food intake after start of solid food was higher in sham rats compared to RYGB rats (SDs 23.3±0.3 vs. 20.5±0.8 grams/24hr, p<0.001; ZDFs 31.5±0.4 vs. 20.8±0.4 grams/ 24hr; p<0.001). The reduction of food intake mirrored a decrease in 24-hr dry fecal weight (S1 Fig.).

### Blood glucose and insulin

In normoglycemic SD rats, there was no difference between RYGB and SHAM groups in steady state non-fasting plasma glucose and insulin concentration throughout the entire study period. At nine weeks of age, when surgeries were performed, ZDF rats exhibited elevated non-fasting plasma glucose and insulin levels relative to SD rats (Fig. 1C and 1D). Normally, male ZDF rats would show an increase in blood glucose with a significant decrease of plasma insulin from nine to thirteen weeks of age [26]. However, consistent with our unpublished data, laparotomy and gastrointestinal surgery manipulation alone (i.e. SHAM operation in the current study) delayed the time course for the development of hyperglycemia in ZDF rats. Surgery and the initial post-operative caloric restriction (liquid diet) reduced plasma glucose in both SHAM and RYGB groups (Fig. 1C). While the ZDF-RYGB group remained normoglycemic till the end of the study, plasma glucose of the ZDF-SHAM group increased (202±11 vs. 300±44mg/dL for ZDF-RYGB and ZDF-SHAM respectively, p = 0.001; Fig. 1C). There was also a significant difference in plasma insulin between the two ZDF groups. ZDF-SHAM rats had a significant increase in plasma insulin at POD14 and POD28 indicating a deterioration of insulin resistance (Fig. 1D). Plasma insulin of the ZDF-RYGB group decreased on POD3 and remained low until completion of the study (Fig. 1D). Together, these data suggest improved glucose homeostasis in ZDF rats after RYGB surgery. The data also highlight the weight-independent benefits of RYGB as the reduction in insulin and glucose levels occurred rapidly and before any significant weight loss; these values remained low compared to pre-operative levels despite the ZDF-RYGB rats recovering their pre-operative weight by POD28.

### Plasma GIP, PYY and GLP-1

**Fig 2:**
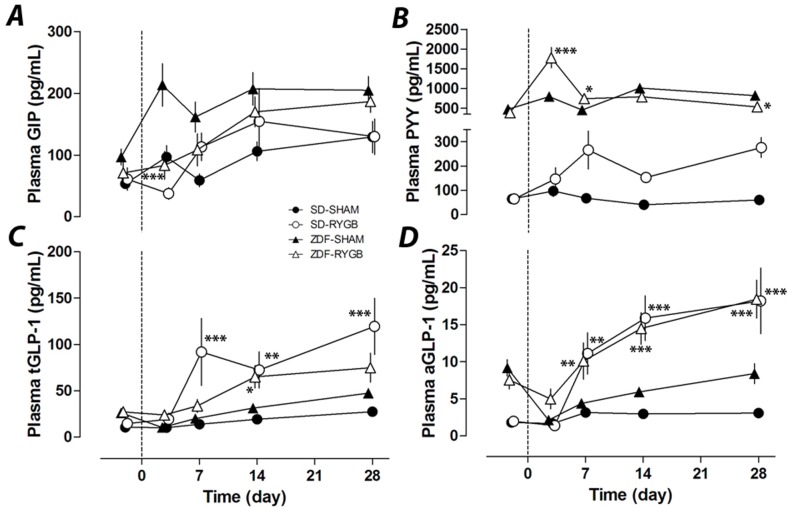
Effects of RYGB on plasma GIP, PYY, total GLP-1 and active GLP-1. RYGB or sham surgeries were performed on day 0. Plasma samples were taken on indicated days up to 28 days. Non-fasting plasma GIP (***A***), PYY (***B***), total GLP-1 (***C***) and active GLP-1 (***D***) were measured on days −2, 3, 7, 14 and 28. * P<0.05, **P<0.01, ***P<0.001 vs. the corresponding sham group.

Despite a 60% decrease in plasma GIP in RYGB rats of both strains at POD3 (38±28 vs. 97±23pg/mL, SD-RYGB vs. SD-SHAM, p = 0.24; 83±21 vs. 213±23pg/mL, ZDF-RYGB vs. ZDF-SHAM, p<0.001), GIP levels subsequently increased and were not significantly different between groups after this (Fig. 2A). SD rats had a low baseline level for plasma PYY. While there was no change in the SD-SHAM group, the SD-RYGB group had a slight increase in plasma PYY from POD 7 to 28 (Fig. 2B). While still on liquid diet, the ZDF-RYGB group had a large but transient spike in plasma PYY at POD 3 (2.3 fold vs. ZDF-SHAM, p<0.001). Plasma PYY in ZDF-RYGB animals did not increase between POD7 and POD28 (Fig. 2B). Non-fasting active and total GLP-1 levels were both elevated in SD-RYGB rats with significant differences from POD 7 onwards (Fig. 2C-D). RYGB surgery also increased plasma GLP-1 in ZDF rats from POD14 (for total GLP-1) and POD7 (active GLP-1) to levels similar to those of SD-RYGB rats. This was especially striking for the active component (Fig. 2C-D).

### Plasma and fecal total bile acids and bile acid composition

**Fig 3:**
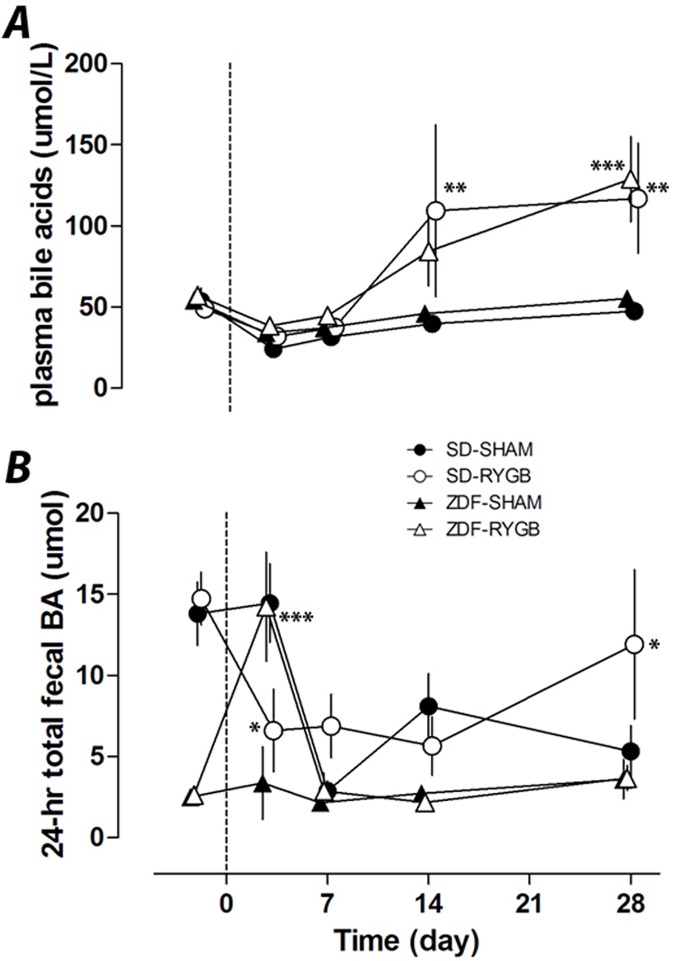
Effects of RYGB on plasma and fecal bile acids. RYGB or sham surgeries were performed on day 0. Plasma and fecal samples were collected on indicated days up to 28 days. Non-fasting plasma total bile acids (***A***) and 24-h total fecal bile acid excretion (***B***) were measured on days −2, 3, 7, 14 and 28. * P<0.05, **P<0.01, ***P<0.001 vs. the corresponding sham group.

Similar to previous reports, RYGB increased plasma total BA. At POD28 plasma BA levels were significantly elevated in SD-RYGB (117±17 vs. 47±14μmol/L in SD-SHAM rats, p<0.01) and in ZDF-RYGB rats (129±13 vs. 56±14μmol/L in ZDF-SHAM, p<0.001) (Fig. 3A). The serum levels were similar between both species irrespective of changes in glucose homeostasis. These changes also did not happen immediately after surgery and are hence unlikely to contribute to the weight-independent improvements in glucose levels noted above. In both rat strains, plasma total BAs were similar for the first week with increased levels developing after 14 days.

ZDF rats had lower baseline levels of total fecal BAs compared to SD rats, and showed a different pattern of change after surgery. Total fecal BA excretion did not change in ZDF-SHAM rats but ZDF-RYGB rats had a transient but marked spike (4.2-fold increase, p<0.001) in fecal BA levels on POD3. Fecal BA excretion was not different between ZDF groups at any other time point (Fig. 3B). The SD-RYGB and SD-SHAM groups had decreased total fecal BA during the study period, although in SD-RYGBs this decrease occurred earlier and on POD3 total fecal BA were less in SD-RYGB than SD-SHAM group, but higher by POD28 (Fig. 3B). Similar changes were observed with weight-normalized fecal BA concentration (S1 Fig.).

To further understand the impact of RYGB on BA metabolism, we analyzed POD3 fecal BA composition in ZDF rats (the time point where there was a significant increase in fecal BA contents after RYGB, without change in serum levels) (Table 1); and at POD28 (the time point where there was a significant increase in plasma BA contents after RYGB) plasma and fecal BA composition in both ZDF (Table 2) and SD rats (S1 Table). On POD3 when fecal BA levels in ZDF-RYGB rats peaked, there was increased excretion of both primary and secondary BAs vs. shams with a slightly higher ratio of CA to CDCA derived BAs in ZDF-RYGB compared to that of ZDF-SHAM (Table 1). ZDF-RYGB rats tended to have higher conjugated (median: 0.81% and 4.32% for sham and RYGB, respectively; p = 0.01) and primary (median: 70.80% and 79.03% for sham and RYGB, respectively; p = 0.03) BAs in feces.

**Table 1:** Fecal bile acid profile on POD3 in ZDF rats.

	**Fecal BA (μg), day 3**		
	**SHAM**	**RYGB**	***P value***
***Unconjugated primary BA***			
CDCA	ND (ND-0.12)	6 (0.59–19)	*<0*.*001*
αMCA	1.9 (1.1–22)	31 (5.3–65)	*0*.*001*
βMCA	4.1 (21–242)	144 (31–200)	*0*.*145*
CA	5.6 (ND-203)	103 (12–250)	*0*.*013*
***Conjugated primary BA***			
T-CDCA	ND (ND-2.3)	1.2 (0.28–11)	*0*.*005*
G-CDCA	ND	ND (ND-0.9)	*0*.*11*
T-aMCA	ND (ND-8.6)	6.1 (ND-92)	*0*.*005*
T-CA	0.93 (ND-44)	6.8 (0.97–56)	*0*.*024*
G-CA	ND	ND (ND-0.70)	*0*.*013*
***Unconjugated secondary BA***			
LCA	1.6 (0.94–4)	6.4 (0.59–22)	*0*.*016*
DCA	28 (5.6–87)	92 (14–143)	*0*.*02*
UDCA	0.72 (0.43–4.7)	2.1 (0.71–17)	*0*.*013*
***Conjugated secondary BA***			
T-LCA	ND	ND (ND-0.74)	*0*.*218*
G-LCA	ND	0.67 (ND-2.5)	*<0*.*001*
T-DCA	ND (ND-0.92)	0.73 (0.026–9.9)	*0*.*002*
G-DCA	ND	0.31 (ND-1.4)	*0*.*007*
T-UDCA	ND	0.22 (ND-2.5)	*0*.*013*
G-UDCA	ND	ND (ND-1.0)	*0*.*218*
*CA/CDCA derived ratio*	0.79 (0.53–2.96)	1.09 (0.85–1.51)	*0*.*061*

Data presented as Median (range); ND: not detectd

**Table 2:** Plasma and fecal bile acid profile on POD28 in ZDF rats.

	**Plasma BA (ng/mL), day 28**			**Fecal BA (μg), day 28**		
	
	**SHAM**	**RYGB**	***P value***	**SHAM**	**RYGB**	***P value***
***Unconjugated Primary BA***						
CDCA	14 (ND-270)	260 (20–1107)	*0*.*002*	0.085 (ND-19)	ND (ND-2.5)	*0*.*49*
aMCA	8 (ND-44)	108 (15–452)	*0*.*002*	ND (ND-81)	3.7 (ND-27)	*0*.*212*
bMCA	0.86 (ND-9.83)	36 (3–71)	*<0*.*001*	86 (44–435)	103 (42–194)	*0*.*805*
CA	223 (17–1170)	717 (234–1812)	*0*.*009*	15 (4.5–214)	4.4 (1.3–111)	*0*.*119*
***Conjugated primary BA***						
T-CDCA	2 (ND-48)	27 (9–89)	*0*.*002*	1.7 (0.38–11)	1.9 (0.72–3.6)	*0*.*712*
G-CDCA	ND (ND-12)	ND (ND-23)	*0*.*175*	ND	ND	*NA*
T-aMCA	ND	17 (5–85)	*<0*.*001*	ND (ND-9.1)	ND (ND-11)	*0*.*614*
T-CA	42 (14–146)	119 (45–267)	*0*.*027*	6.6 (4.7–63)	3 (ND-8.5)	*0*.*009*
G-CA	ND	ND	*NA*	ND	ND	*NA*
***Unconjugated secondary BA***						
LCA	ND	ND	*NA*	6.5 (0.97–44)	4.7 (0.32–9.1)	*0*.*796*
DCA	ND (ND-54)	15 (0.7–87)	*0*.*012*	22 (6.2–380)	24 (5.8–51)	*0*.*937*
UDCA	ND (ND-27)	37 (ND-109)	*0*.*002*	2.2 (ND-44)	1.8 (ND-7.2)	*0*.*796*
***Conjugated secondary BA***						
T-LCA	ND	ND	*NA*	ND	ND	*NA*
G-LCA	4 (ND-258)	35 (7–86)	*0*.*009*	ND (ND-3.5)	ND	*0*.*088*
T-DCA	ND (ND-7)	0.11 (ND-4)	*0*.*14*	ND (ND-2.9)	ND (ND-0.084)	*0*.*119*
G-DCA	ND	ND	*NA*	ND (ND-1.4)	ND	*0*.*088*
T-UDCA	ND	ND	*NA*	ND (ND-2.9)	ND	*0*.*088*
G-UDCA	ND	ND	*NA*	ND	ND	*NA*
*CA*:*CDCA derived ratio*	6.90 (2.14–10.31)	1.97 (1.05–3.92)	*0*.*002*	0.5 (0.34–1.4)	0.33 (0.21–0.82)	*0*.*108*

Data presented as Median (range); ND: not detected; NA: not applied

At POD28, both primary and secondary plasma BAs were increased in ZDF-RYGB animals (Table 2) and had a trend to be higher in SD-RYGB (S1 Table). However, the ratio of CA-derived BAs (CA, T-CA, G-CA, DCA, T-DCA and G-DCA) to CDCA-derived BAs (CDCA, αMCA, βMCA, T-CDCA, G-CDCA, T-αMCA, LCA, T-LCA, and G-LCA) in plasma decreased in RYGB animals for both ZDF and SD rats. Of particular note, RYGB surgery reduced the plasma CA:CDCA ratio in ZDF rats from 6.9 to 1.97 (p = 0.002), which was very similar to the plasma CA:CDCA ratio of 1.86 in SD-SHAM animals (S1 Table). Fecal CA:CDCA ratios also decreased slightly in the RYGB groups compared to the SHAM groups on POD28 (Table 2 and S1 Table), suggesting a post-operative alteration of the BA pool composition.

### Tissue gene expression

To further understand how RYGB surgery changed BA metabolism, we measured expression of hepatic genes involved in BA biosynthesis and intestinal genes involved in BA enterohepatic circulation with quantitative RT-PCR. All tissue samples were taken on POD28. Comparisons between RYGB and SHAM groups (fold changes) are presented in Table 3. Due to small numbers of animals used for gene expression and variations within groups, some changes failed to reach statistic significance. Individual animal data and relative tissue expression levels can be found in S2–S5 Figs.

**Table 3:** Hepatic and intestinal mRNA expression of BA pathway enzymes by quantitative RT-PCR.

	**Fold change, RYGB vs. SHAM**														
	**ZDF rat**								**SD rat**						
**Gene**	**BP**	**Rx**	**Cm**	**TI**	**AC**	**DC**	**Liver**		**BP**	**Rx**	**Cm**	**TI**	**AC**	**DC**	**Liver**
Cyp7a1	NQ	NQ	1.12	3.48	NQ	NQ	−1.35		NQ	NQ	NQ	NQ	NQ	NQ	1.01
Cyp27a1	1.48	−1.94	−1.31	−1.65	−1.77	−1.17	−1.36		−1.15	−2.91*	−3.31*	-1.86*	−1.79*	−2.87	−1.24
Cyp8b1	−1.12	1.30	1.05	1.43	−1.37	−1.39	−2.45		NQ	NQ	NQ	NQ	−3.97*	-3.32*	-2.62*
Nr1h4 (Fxr)	−1.42	−2.31	1.17	−1.97	−2.04	−1.04	−2.51		−1.41	−1.72*	−1.41	−1.27	−1.43	-2.05*	-1.63*
Nr0b2 (Shp)	2.34	−5.88	−1.30	1.23	-4.44*	−1.14	1.06		1.95	−4.65*	1.38	-4.10*	−1.66	-2.11*	-2.33*
Nr1i2 (Pxr)	−1.06	−1.47	1.03	−1.40	−1.42	−1.02	-2.63*		1.05	−1.36	−1.24	-1.69*	−1.13	-1.85*	−1.44
Fgf15	NQ#	NQ	1.67	1.09	−5.36	−2.59	NQ		NQ#	NQ	2.89	-9.14*	−2.45	1.12	NQ
Slc10a1 (NTCP)	1.63	−1.55	1.20	4.56	2.28	−1.10	−1.12		NQ	NQ	NQ	NQ	−1.88	NQ	−1.57
Slc10a2 (Asbt)	1.47	−1.07	2.82	−2.22	−4.55	NQ	−1.47		−1.55	1.25	1.96	−1.31	−1.51	NQ	−1.20
Abcb11 (Bsep)	−1.18	−2.03	1.10	−1.20	−1.83	1.77	−1.62		−2.04	−2.49*	−2.01	-2.65*	−1.17	−1.71	-2.08*
Slc51a (Ost1α)	−1.27	−1.41	1.07	−1.90	−5.15	−1.19	1.20		−1.62	−1.27	−1.41	−1.54	−4.42*	-4.01*	-3.38*
Slc52b (Ost1β)	1.10	−1.25	1.28	−2.30	−2.06	1.33	−1.33		1.05	1.06	1.23	−1.63	−2.95	−1.45	−2.33

NQ: very low expression, not quantified

* RYGB group is significantly different from SHAM group, P<0.05 (see the method section more detailed method for statistics)

# fold changes couldn’t be calculated because SHAM groups had very low expression; please see S2–S5 Figs. for individual animal data

### Liver

Hepatic expression of BA-regulatory genes Fxr (Nr1h4) was decreased in both ZDF-RYGB and SD-RYGB vs. sham controls (Table 3, S3 Fig.). Expression of Pxr (Nr1i2) was also decreased in ZDF-RYGB (Table 3, S3 Fig.). There were no changes in ZDF-RYGB but decreased expression of Shp (Nr0b2) in SD-RYGB (Table 3, S3 Fig.). There were no changes in the expression of Car (Nr1i3), Lxrα (Nr1h3) and Lxrβ (Nr1h2) (data not shown). For liver enzymes involved in BA biosynthesis both ZDF-RYGB and SD-RYGB rats had similar expression levels of Cyp7a1 and Cyp27a1 but decreased expression of Cyp8b1 (−2.45 and −2.62 fold for ZDF-RYGB and SD-RYGB, respectively) compared to corresponding sham groups (Table 3 and S2 Fig.). With regard to genes involved in hepatic BA uptake, Ntcp (Slc10a1) expression did not change in ZDF-RYGB but had a trend to be lower in SD-RYGB vs. sham controls (Table 3 and S4 Fig.). There was no decrease in the expression of OATPs (unpublished data). The major hepatic BA export transporter Bsep (Abcb11) tended to be down-regulated in both SD-RYGB and ZDF-RYGB rats (Table 3 and S5 Fig.).

### Intestine

RYGB-induced changes in plasma and fecal BAs may be a consequence of alterations in intestinal BA absorption via the apical sodium bile acid transporter (Asbt). Although predominantly expressed in TI, we found low level mRNA expression of Asbt in the distal jejunum of sham rats (orthotopic Cm limb; S4 Fig.). RYGB showed a trend towards enhanced expression of Asbt in Cm limb in both SD and ZDF rats, but this increase did not reach statistic significance (Table 3 and S4 Fig.). There was no change in Asbt expression in the TI but a trend towards lower levels in AC of ZDF-RYGB rats (Table 3 and S4 Fig.). Enterocyte BA exporting transporters, Ostα (Slc51a) and Ostβ (Slc51b), were also predominantly expressed in TI and distal jejunum of sham rats with relatively low expression in the rest of the intestine (S5 Fig.). Both Ostα and Ostβ expression were down-regulated in TI and AC in SD- and ZDF-RYGB groups (Table 3 and S5 Fig.). In rat intestine, Fxr showed topographic variation with highest levels of expression in the terminal ileum followed by distal jejunum, ascending colon and descending colon (S3 Fig.). Fxr mRNA was down-regulated in AC of ZDF-RYGB rats and DC of SD-RYGB rats (Table 3, S3 Fig.). Although expressed at relatively low levels in the orthotopic Rx limb of sham rats, Fxr expression was further reduced after RYGB in both ZDF and SD rats.

For BA (Fxr-mediated) regulated genes, Shp expression was increased in the BP limb and decreased in the Rx limb and AC or DC for both RYGB groups (Table 3, S3 Fig.), although some changes didn’t reach statistical significance after accounting for multiple testing using the current method. Fgf15 was mainly expressed in TI and the distal part of the jejunum (Cm limb) with large variations in all groups (S4 Fig.). While almost undetectable in the duodenum of sham groups, Fgf15 was significantly increased in the BP limb of both RYGB groups (Table 3, S4 Fig.).

## Discussion

Studies consistently report increased circulating BAs after RYGB surgery in humans although underlying mechanisms for this change and the relevance to the improved metabolic profile seen after surgery are unclear [10–14,27]. Despite suggestions that post-RYGB increases in plasma BAs mediate early remission of T2DM, a clear cause-effect relationship has not been established. In addition, the mechanism for increased post-surgery circulating BAs is unclear. In this study, we developed RYGB models in both non-obese normoglycemic SD rats and obese hyperglycemic ZDF rats and reproduced changes seen in human RYGB patients including improvement in glucose metabolism, and increased plasma GLP-1, PYY and total BAs. Using those rodent models, we were able to further explore RYGB-mediated changes in BA metabolism.

### Slow onset of increase in plasma bile acids

In both SD and ZDF rats, the rise in plasma BAs was seen at POD14 and not in the immediate/early post-operative period. Therefore, the RYGB-induced increase in plasma total BAs is not an immediate consequence of anatomical changes after surgery but likely develops with postoperative adaptations. Most reports published to date for both humans and animals compare circulating BA levels at a single time point weeks or months post-surgery [10–14,27–29]. In a small longitudinal, prospective pilot study (five obese patients), Ahmad et al. demonstrated that changes in postprandial circulating BAs were time-dependent post-RYGB [30]. Although there were no early changes in serum BAs, a significant increase in postprandial BAs developed by 40 weeks after RYGB. Considering the differences in lifespan, development and physiology between rodents and humans [31], our data are consistent with these findings in humans. In another study, the time course for post-surgery increases in plasma BAs did not match the time course for marked increases in GLP-1 and PYY and improved glycemic control [14], which is consistent with our findings. Together, these data suggest that RYGB-induced changes in plasma BAs are unlikely to mediate early and weight-independent improvements in glucose homeostasis although they may contribute to sustained improvements in the later post-operative stage.

### Potential mechanisms for RYGB mediated changes in bile acid metabolism

Because both SD-RYGB and ZDF-RYGB groups had increased plasma BAs, we predicted a common mechanism for elevated BAs between the two models and focused our investigation on possible changes in liver BA production and enterohepatic BA recirculation, in particular on FXR-related pathways [32]. Recently, Ryan et al. have shown that the absence of FXR, via which BAs exert metabolic effects, decreases the benefit of bariatric surgery on weight loss and glucose tolerance [33]. Changes in circulating BAs might be caused by an increase in hepatic BA production or a decrease in hepatic BA uptake. RYGB surgery caused decreases in hepatic Fxr expression in both rat strains and Shp expression in SD rats which could potentially lead to increases in hepatic Cyp7a1 and Cyp8b1 expressions (two key enzymes in BA biosynthesis) and hepatic BA production. However, both RYGB groups had reduced liver Cyp8b1 expression with no change in Cyp7a1 expression. Using pooled serum samples, we detected no changes in 7alpha-hydroxy-4-cholesten-3-one (C4), a marker for liver BA synthesis, after RYGB in both strains (data not shown). Altogether, our data didn’t suggest an increase in hepatic BA production after RYGB.

At physiological levels hepatic BA uptake from portal blood functions well below saturation point [34], hence an increase of circulating BA via inhibition of hepatic uptake would require significant changes in carrier-mediated BA transport systems [35]. Again, our data on mediators of hepatic BA uptake (e.g. Ntcp) did not show significant or consistent changes in SD and ZDF rats following RYGB.

Kohli et al demonstrated that ileal interposition, in which a segment of ileum was relocated to the distal part of duodenum, led to early reabsorption of BAs in the small intestine and an increase in circulating BAs in rats [36]. Similar proximal reabsorption of BA was observed in GATA4 knockout mice, which had increased Asbt expression in jejunum and elevated serum BAs [37,38]. We hypothesized that altered reabsorption of BAs in proximal small intestine could be another potential mediator of increased circulating BAs post-RYGB. RYGB surgery dramatically alters intestinal anatomy and exposure to bile, pancreatic enzymes and food, which could plausibly alter BA reabsorption. The BP limb had bile but no food while the Roux limb had food but no bile. Since BAs needed to be absorbed into enterocytes to induce expression of Shp, Shp expression was significantly reduced in the Roux limb where BA exposure was precluded. Despite generally lower Fxr mRNA expression following RYGB in both SD and ZDF rats, the BP limb had increased Shp expression compared to the corresponding intestinal segment in the sham groups. In BP limb, Fgf15 expression, regulated by BAs and Fxr, was also up-regulated but Asbt expression was very low and unchanged suggesting increased Asbt-independent BA absorption in that region of bowel. RT-qPCR analyses also demonstrated a trend towards increased Asbt mRNA in the Cm limb of both SD-RYGB and ZDF-RYGB, suggesting potentially more BA reabsorption in that part of jejunum- similar to observations in GATA4 knockout mice [37,38]. Interestingly, expression of GATA4 in the Cm limb of ZDF-RYGB rats in our study was significantly down-regulated (unpublished data). In addition, some changes in Shp and Fgf15 expression also suggested potentially less BA reabsorption in terminal ileum and/or ascending colon. Altogether, the gene expression pattern suggested increased BA reabsorption in proximal small intestine but didn’t explain why an early absorption of BAs could increase circulating BA levels. Potential mechanisms are currently being investigated in our lab.

### Effects of RYGB on plasma and fecal bile acid composition

Several groups report changes in serum BA composition after bariatric surgeries in humans and animals [11,39,40] but results vary from study to study. In the current study, we noticed a significant decrease in the plasma ratio of CA derived BAs (e.g. conjugated and unconjugated CA and DCA) to CDCA derived BAs (e.g. conjugated and unconconjugated CDCA, αMCA, βMCA and LCA). That ratio change could be caused by preferential BA reabsorption via intestinal transporters such as Asbt [38,41,42]. If that was the only mechanism, an increased ratio of CA:CDCA-derived BA in feces (more absorption of CDCA) might be expected. On the contrary, the fecal ratio of CA:CDCA derived BAs also decreased slightly in RYGB groups. Therefore, we hypothesized that the liver might produce relatively more CDCA than CA post-surgery. This hypothesis was supported by a significant decrease of hepatic Cyp8b1 expression in RYGB groups. Cyp8b1, also known as sterol 12-alpha-hydroxylase, is an endoplasmic reticulum membrane protein that catalyzes the conversion of 7 alpha-hydroxy-4-cholesten-3-one into 7-alpha,12-alpha-dihydroxy-4-cholesten-3-one. The balance between these two intermediates of BA synthesis determines the relative production of the two primary BAs, cholic acid and chenodeoxycholic acid. Changes in ratio of CA:CDCA under different metabolic conditions have been reported previously. For example, significantly increased CA:CDCA ratio has been reported for multiple diabetic animal models [43–45]. Importantly, correction of hyperglycemia decreased that ratio [46,47]. Similar findings were reported in human insulin-resistant patients [48,49]. The decreased CA:CDCA after RYGB in our study is consistent with this and may be a consequence of metabolic benefits of bariatric surgery. Although not examined in this study, the effects of microbiome on bile acid metabolism should also be considered[50].

### Possible early changes in post-surgery BA exposure in distal intestines

In addition to plasma BA changes, we report post-RYGB fecal BA excretion patterns in rats. Despite a doubling of plasma BAs at the end of the study, there was no reduction of fecal BA excretion in either SD-RYGB or ZDF-RYGB compared to sham groups. Interestingly, there was a significant difference between SD and ZDF rats on POD3. While SD-RYGB tended to have lower 24-hour total fecal BAs, ZDF-RYGB rats had a transient 4.2-fold increase in fecal BA excretion vs. ZDF-SHAM. This spike occurred in parallel with a spike in plasma PYY levels which might be a consequence of an increased colonic load of BAs exerting effects on enteroendocrine cells in the distal bowel. Direct administration of BAs to the colon induces secretion of peptides (GLP-1 and PYY) from L-cells [51] while increasing the delivery of BAs to the distal intestine with BA sequestrants or Asbt inhibitors not only increases plasma GLP-1 and PYY but also significantly improves glycemic control in ZDF rats [21,22]. Therefore the transient but marked early change in fecal BA excretion in ZDF-RYGB rats could have significant metabolic impacts, and possibly contribute to the early and weight independent metabolic benefits. It will be interesting to find out if such changes are associated with diabetic animals only. More importantly, this needs to be verified in human patients.

In addition to their critical role in lipid digestion and absorption, BAs also act as endocrine factors on gut peptide secretion [17], glucose and lipid metabolism [52,53], and energy expenditure [19], all of which may contribute to weight loss and remission of T2DM following bariatric surgery. Although the current study cannot establish causality between BAs and metabolic effects of RYGB, our data provide unique information on post-RYGB BA metabolism which otherwise can’t be obtained easily from human studies. The current observations suggest that plasma BAs are unlikely to be critical mediators of early remission of T2DM after RYGB. While there is no indication for increased hepatic BA production, proximal reabsorption of BAs in small intestine prior to the terminal ileum may be increased after RYGB however it is unclear if the overall intestinal BA absorptive power is increased and can account for the increase in serum BA. Furthermore, a transient increase in fecal BA excretion (increased colon exposure) may be a mechanism for RYGB-mediated early improvement in glucose metabolism.

## Supporting Information

S1 Fig Fecal weight and day 3 fecal bile acid concentration in ZDF rats. (PDF)

S2 Fig Tissue mRNA expression: Cyp7a1, Cyp8b1 and Cyp27a1. (PDF)

S3 Fig Tissue mRNA expression: Fxr, Shp and Pxr. (PDF)

S4 Fig Tissue mRNA expression: Fgf15, Ntcp and Asbt. (PDF)

S5 Fig Tissue mRNA expression: Abcb11, Ostα and Ostβ. (PDF)

S1 Table Plasma and fecal bile acid profile on POD28 in SD rats. (PDF)

## References

[1] Buchwald H, Rucker RD. The history of metabolic surgery for morbid obesity and a commentary. World J Surg 1981 Nov;5(6):781–7. 7043911 10.1007/BF01657963

[2] Sjostrom L. Review of the key results from the Swedish Obese Subjects (SOS) trial—a prospective controlled intervention study of bariatric surgery. J Intern Med 2013 Mar;273(3):219–34. 10.1111/joim.12012 23163728

[3] Pories WJ, Mehaffey JH, Staton KM. The surgical treatment of type two diabetes mellitus. Surg Clin North Am 2011 Aug;91(4):821–36, viii. 10.1016/j.suc.2011.04.008 21787970

[4] Schauer PR, Kashyap SR, Wolski K, Brethauer SA, Kirwan JP, Pothier CE, et al Bariatric surgery versus intensive medical therapy in obese patients with diabetes. N Engl J Med 2012 Apr 26;366(17):1567–76. 10.1056/NEJMoa1200225 22449319 PMC3372918

[5] Kashyap SR, Gatmaitan P, Brethauer S, Schauer P. Bariatric surgery for type 2 diabetes: weighing the impact for obese patients. Cleve Clin J Med 2010 Jul;77(7):468–76. 10.3949/ccjm.77a.09135 20601620 PMC3102524

[6] Neff KJ, O'Shea D, le Roux CW. Glucagon like peptide-1 (GLP-1) dynamics following bariatric surgery: a Signpost to a new frontier. Curr Diabetes Rev 2013 Mar 1;9(2):93–101. 23230996 10.2174/1573399811309020001

[7] Rhee NA, Vilsboll T, Knop FK. Current evidence for a role of GLP-1 in Roux-en-Y gastric bypass-induced remission of type 2 diabetes. Diabetes Obes Metab 2012 Apr;14(4):291–8. 10.1111/j.1463-1326.2011.01505.x 21951387

[8] Saeidi N, Meoli L, Nestoridi E, Gupta NK, Kvas S, Kucharczyk J, et al Reprogramming of intestinal glucose metabolism and glycemic control in rats after gastric bypass. Science 2013 Jul 26;341(6144):406–10. 10.1126/science.1235103 23888041 PMC4068965

[9] Stearns AT, Balakrishnan A, Tavakkolizadeh A. Impact of Roux-en-Y gastric bypass surgery on rat intestinal glucose transport. Am J Physiol Gastrointest Liver Physiol 2009 Nov;297(5):G950–G957. 20501442 10.1152/ajpgi.00253.2009 PMC2777457

[10] Kohli R, Bradley D, Setchell KD, Eagon JC, Abumrad N, Klein S. Weight loss induced by Roux-en-Y gastric bypass but not laparoscopic adjustable gastric banding increases circulating bile acids. J Clin Endocrinol Metab 2013 Apr;98(4):E708–E712. 10.1210/jc.2012-3736 23457410 PMC3615197

[11] Patti ME, Houten SM, Bianco AC, Bernier R, Larsen PR, Holst JJ, et al Serum bile acids are higher in humans with prior gastric bypass: potential contribution to improved glucose and lipid metabolism. Obesity (Silver Spring) 2009 Sep;17(9):1671–7. 10.1038/oby.2009.102 19360006 PMC4683159

[12] Pournaras DJ, le Roux CW. Are bile acids the new gut hormones? Lessons from weight loss surgery models. Endocrinology 2013 Jul;154(7):2255–6. 10.1210/en.2013-1383 23794408

[13] Simonen M, Dali-Youcef N, Kaminska D, Venesmaa S, Kakela P, Paakkonen M, et al Conjugated bile acids associate with altered rates of glucose and lipid oxidation after Roux-en-Y gastric bypass. Obes Surg 2012 Sep;22(9):1473–80. 10.1007/s11695-012-0673-5 22638681 PMC4426904

[14] Steinert RE, Peterli R, Keller S, Meyer-Gerspach AC, Drewe J, Peters T, et al Bile acids and gut peptide secretion after bariatric surgery: A 1-year prospective randomized pilot trial. Obesity (Silver Spring) 2013 Dec;21(12):E660–E668. 10.1002/oby.20522 23804517

[15] de Aguiar Vallim TQ, Tarling EJ, Edwards PA. Pleiotropic roles of bile acids in metabolism. Cell Metab 2013 May 7;17(5):657–69. 10.1016/j.cmet.2013.03.013 23602448 PMC3654004

[16] Fiorucci S, Mencarelli A, Palladino G, Cipriani S. Bile-acid-activated receptors: targeting TGR5 and farnesoid-X-receptor in lipid and glucose disorders. Trends Pharmacol Sci 2009 Nov;30(11):570–80. 10.1016/j.tips.2009.08.001 19758712

[17] Katsuma S, Hirasawa A, Tsujimoto G. Bile acids promote glucagon-like peptide-1 secretion through TGR5 in a murine enteroendocrine cell line STC-1. Biochem Biophys Res Commun 2005 Apr 1;329(1):386–90. 15721318 10.1016/j.bbrc.2005.01.139

[18] Pournaras DJ, Glicksman C, Vincent RP, Kuganolipava S, Alaghband-Zadeh J, Mahon D, et al The role of bile after Roux-en-Y gastric bypass in promoting weight loss and improving glycaemic control. Endocrinology 2012 Aug;153(8):3613–9. 10.1210/en.2011-2145 22673227 PMC3404349

[19] Watanabe M, Houten SM, Mataki C, Christoffolete MA, Kim BW, Sato H, et al Bile acids induce energy expenditure by promoting intracellular thyroid hormone activation. Nature 2006 Jan 26;439(7075):484–9. 16400329 10.1038/nature04330

[20] Smushkin G, Sathananthan M, Piccinini F, Dalla MC, Law JH, Cobelli C, et al The effect of a bile acid sequestrant on glucose metabolism in subjects with type 2 diabetes. Diabetes 2013 Apr;62(4):1094–101. 10.2337/db12-0923 23250357 PMC3609563

[21] Chen L, McNulty J, Anderson D, Liu Y, Nystrom C, Bullard S, et al Cholestyramine reverses hyperglycemia and enhances glucose-stimulated glucagon-like peptide 1 release in Zucker diabetic fatty rats. J Pharmacol Exp Ther 2010 Jul;334(1):164–70. 10.1124/jpet.110.166892 20413600

[22] Chen L, Yao X, Young A, McNulty J, Anderson D, Liu Y, et al Inhibition of apical sodium-dependent bile acid transporter as a novel treatment for diabetes. Am J Physiol Endocrinol Metab 2012 Jan 1;302(1):E68–E76. 10.1152/ajpendo.00323.2011 21934041

[23] Meissner M, Herrema H, van Dijk TH, Gerding A, Havinga R, Boer T, et al Bile acid sequestration reduces plasma glucose levels in db/db mice by increasing its metabolic clearance rate. PLoS One 2011;6(11):e24564 10.1371/journal.pone.0024564 22087215 PMC3210115

[24] Benjamini Y, Hochberg Y. Controlling the False Discovery Rate: A Practical and Powerful Approach to Multiple Testing. Journal of the Royal Statistical Society Series B (Methodological) 1995;57(1):289–300.

[25] Smyth GK. Linear models and empirical bayes methods for assessing differential expression in microarray experiments. Stat Appl Genet Mol Biol 2004;3(article 3). 10.2202/1544-6115.1027 16646809

[26] Peterson RG, Shaw WN, Neel M-A, Little LA, Eichberg J. Zucker Diabetic Fatty Rat as a Model for Non-insulin-dependent Diabetes Mellitus. ILAR J 1990;32(3):16–9. 10.1093/ilar.32.3.13 PMC7793032 34191867

[27] Nakatani H, Kasama K, Oshiro T, Watanabe M, Hirose H, Itoh H. Serum bile acid along with plasma incretins and serum high-molecular weight adiponectin levels are increased after bariatric surgery. Metabolism 2009 Oct;58(10):1400–7. 10.1016/j.metabol.2009.05.006 19570554

[28] Ashrafian H, Li JV, Spagou K, Harling L, Masson P, Darzi A, et al. Bariatric Surgery Modulates Circulating and Cardiac Metabolites. J Proteome Res 2013 Nov 26. 10.1021/pr400748f 24279706

[29] Kohli R, Setchell KD, Kirby M, Myronovych A, Ryan KK, Ibrahim SH, et al A surgical model in male obese rats uncovers protective effects of bile acids post-bariatric surgery. Endocrinology 2013 Jul;154(7):2341–51. 10.1210/en.2012-2069 23592746 PMC3689286

[30] Ahmad NN, Pfalzer A, Kaplan LM. Roux-en-Y gastric bypass normalizes the blunted postprandial bile acid excursion associated with obesity. Int J Obes (Lond) 2013 Dec;37(12):1553–9. 10.1038/ijo.2013.38 23567924 PMC4157126

[31] Sengupta P. The Laboratory Rat: Relating Its Age With Human's. Int J Prev Med 2013 Jun;4(6):624–30. 23930179 PMC3733029

[32] Kim I, Ahn SH, Inagaki T, Choi M, Ito S, Guo GL, et al Differential regulation of bile acid homeostasis by the farnesoid X receptor in liver and intestine. J Lipid Res 2007 Dec;48(12):2664–72. 17720959 10.1194/jlr.M700330-JLR200

[33] Ryan KK, Tremaroli V, Clemmensen C, Kovatcheva-Datchary P, Myronovych A, Karns R, et al FXR is a molecular target for the effects of vertical sleeve gastrectomy. Nature 2014 May 8;509(7499):183–8. 10.1038/nature13135 24670636 PMC4016120

[34] Reichen J, Paumgartner G. Kinetics of taurocholate uptake by the perfused rat liver. Gastroenterology 1975 Jan;68(1):132–6. 1090476

[35] Stieger B. The role of the sodium-taurocholate cotransporting polypeptide (NTCP) and of the bile salt export pump (BSEP) in physiology and pathophysiology of bile formation. Handb Exp Pharmacol 2011;(201):205–59. 10.1007/978-3-642-14541-4_5 21103971

[36] Kohli R, Kirby M, Setchell KD, Jha P, Klustaitis K, Woollett LA, et al Intestinal adaptation after ileal interposition surgery increases bile acid recycling and protects against obesity-related comorbidities. Am J Physiol Gastrointest Liver Physiol 2010 Sep;299(3):G652–G660. 10.1152/ajpgi.00221.2010 20595624 PMC2950688

[37] Battle MA, Bondow BJ, Iverson MA, Adams SJ, Jandacek RJ, Tso P, et al GATA4 is essential for jejunal function in mice. Gastroenterology 2008 Nov;135(5):1676–86. 10.1053/j.gastro.2008.07.074 18812176 PMC2844802

[38] Beuling E, Kerkhof IM, Nicksa GA, Giuffrida MJ, Haywood J, aan de Kerk DJ, et al Conditional Gata4 deletion in mice induces bile acid absorption in the proximal small intestine. Gut 2010 Jul;59(7):888–95. 10.1136/gut.2009.204990 20581237 PMC2981798

[39] Gustafsson U, Benthin L, Granstrom L, Groen AK, Sahlin S, Einarsson C. Changes in gallbladder bile composition and crystal detection time in morbidly obese subjects after bariatric surgery. Hepatology 2005 Jun;41(6):1322–8. 15834935 10.1002/hep.20686

[40] Cummings BP, Bettaieb A, Graham JL, Stanhope KL, Kowala M, Haj FG, et al Vertical sleeve gastrectomy improves glucose and lipid metabolism and delays diabetes onset in UCD-T2DM rats. Endocrinology 2012 Aug;153(8):3620–32. 10.1210/en.2012-1131 22719048 PMC3404344

[41] Kramer W, Stengelin S, Baringhaus KH, Enhsen A, Heuer H, Becker W, et al Substrate specificity of the ileal and the hepatic Na(+)/bile acid cotransporters of the rabbit. I. Transport studies with membrane vesicles and cell lines expressing the cloned transporters. J Lipid Res 1999 Sep;40(9):1604–17. 10484607

[42] Zhang EY, Knipp GT, Ekins S, Swaan PW. Structural biology and function of solute transporters: implications for identifying and designing substrates. Drug Metab Rev 2002 Nov;34(4):709–50. 12487148 10.1081/dmr-120015692

[43] Hassan AS, Subbiah MT, Thiebert P. Specific changes of bile acid metabolism in spontaneously diabetic Wistar rats. Proc Soc Exp Biol Med 1980 Sep;164(4):449–52. 7413665 10.3181/00379727-164-40894

[44] Uchida K, Takase H, Kadowaki M, Nomura Y, Matsubara T, Takeuchi N. Altered bile acid metabolism in alloxan diabetic rats. Jpn J Pharmacol 1979 Aug;29(4):553–62. 537273 10.1254/jjp.29.553

[45] Uchida K, Makino S, Akiyoshi T. Altered bile acid metabolism in nonobese, spontaneously diabetic (NOD) mice. Diabetes 1985 Jan;34(1):79–83. 3964756 10.2337/diab.34.1.79

[46] Ishida H, Yamashita C, Kuruta Y, Yoshida Y, Noshiro M. Insulin is a dominant suppressor of sterol 12 alpha-hydroxylase P450 (CYP8B) expression in rat liver: possible role of insulin in circadian rhythm of CYP8B. J Biochem 2000 Jan;127(1):57–64. 10731667 10.1093/oxfordjournals.jbchem.a022584

[47] Nervi FO, Severin CH, Valdivieso VD. Bile acid pool changes and regulation of cholate synthesis in experimental diabetes. Biochim Biophys Acta 1978 May 25;529(2):212–23. 656452 10.1016/0005-2760(78)90064-4

[48] Haeusler RA, Astiarraga B, Camastra S, Accili D, Ferrannini E. Human insulin resistance is associated with increased plasma levels of 12alpha-hydroxylated bile acids. Diabetes 2013 Dec;62(12):4184–91. 10.2337/db13-0639 23884887 PMC3837033

[49] Andersen E, Karlaganis G, Sjovall J. Altered bile acid profiles in duodenal bile and urine in diabetic subjects. Eur J Clin Invest 1988 Apr;18(2):166–72. 3133222 10.1111/j.1365-2362.1988.tb02408.x

[50] Joyce SA, Shanahan F, Hill C, Gahan CG. Bacterial bile salt hydrolase in host metabolism: potential for influencing astrointestinal microbe-host crosstalk. Gut Microbes 2014 Sep 1;0. 10.4161/19490976.2014.969986 PMC4615832 25483337

[51] Plaisancie P, Dumoulin V, Chayvialle JA, Cuber JC. Luminal glucagon-like peptide-1(7–36) amide-releasing factors in the isolated vascularly perfused rat colon. J Endocrinol 1995 Jun;145(3):521–6. 7636436 10.1677/joe.0.1450521

[52] Nguyen A, Bouscarel B. Bile acids and signal transduction: role in glucose homeostasis. Cell Signal 2008 Dec;20(12):2180–97. 10.1016/j.cellsig.2008.06.014 18634871

[53] Porez G, Prawitt J, Gross B, Staels B. Bile acid receptors as targets for the treatment of dyslipidemia and cardiovascular disease. J Lipid Res 2012 Sep;53(9):1723–37. 10.1194/jlr.R024794 22550135 PMC3413216

