# Supplementary material for: Effect of Roux-en-Y Gastric Bypass Surgery on Bile Acid Metabolism in Normal and Obese Diabetic Rats
Source: PLoS One. 2015 Mar 23;10(3):e0122273. doi: 10.1371/journal.pone.0122273 (PMC4370587; doi:10.1371/journal.pone.0122273)
Supplement: S1 Fig — (PDF) [file pone.0122273.s001.pdf]

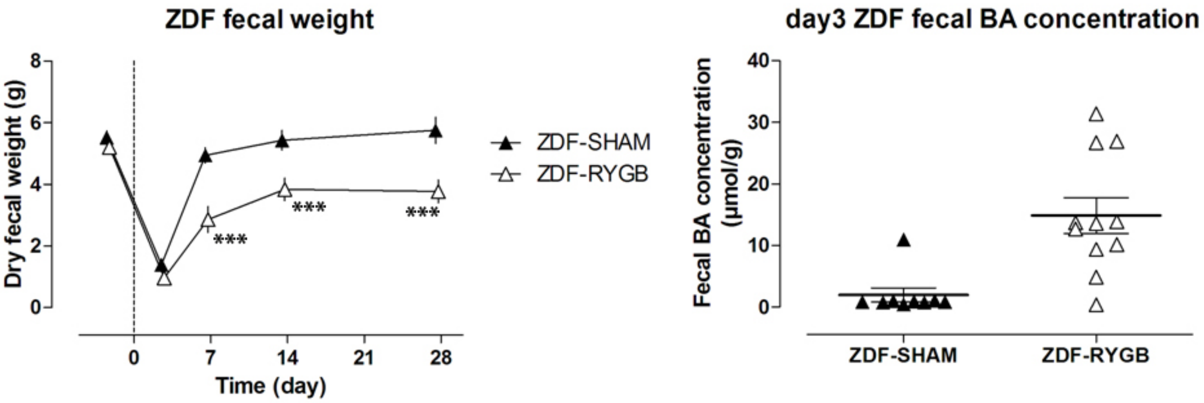

S1-Figure RYGB or sham surgeries were performed on day 0 in ZDF rats. Dry 24-h total fecal weight (***A***) and fecal weight corrected 24-h total fecal bile acid excretion on day 3 (***B***) were measured. ***P<0.001 vs. the sham group.
