# Supplementary material for: Effect of Roux-en-Y Gastric Bypass Surgery on Bile Acid Metabolism in Normal and Obese Diabetic Rats
Source: PLoS One. 2015 Mar 23;10(3):e0122273. doi: 10.1371/journal.pone.0122273 (PMC4370587; doi:10.1371/journal.pone.0122273)
Supplement: S3 Fig — (PDF) [file pone.0122273.s003.pdf]

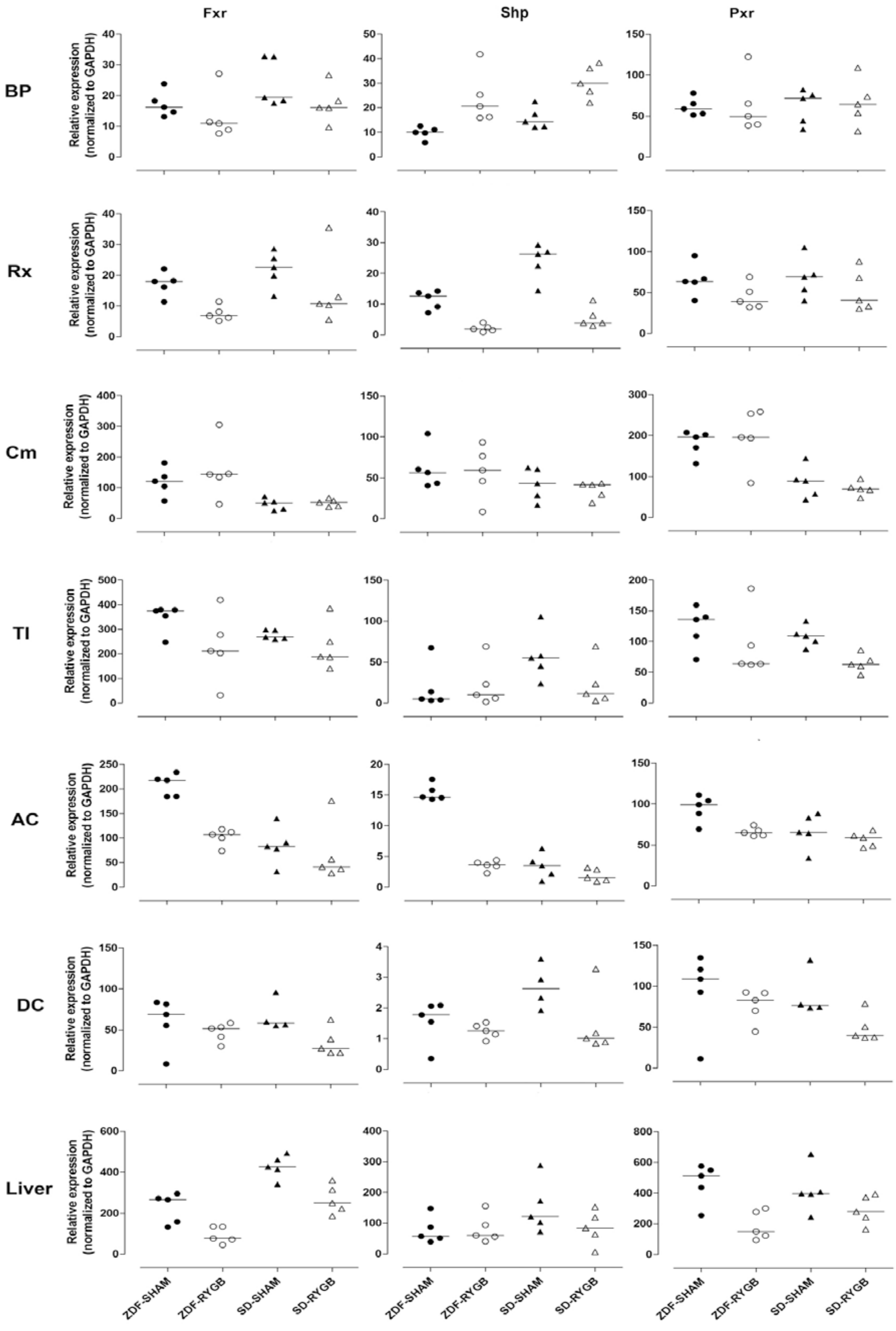

Fxr
Shp
Pxr
BP
Rx
Cm
Tl
AC
DC
Liver
Relative expression (normalized to GAPDH)
ZDF-SHAM
ZDF-RYGB
SD-SHAM
SD-RYGB

S4_Figure Tissue samples were collected on day 28 without fasting from biliopancreatic (BP) limb, Roux (Rx) limb, common (Cm) limb, terminal ileum (TI), ascending colon (AC), descending colon (DC) and liver in RYGB rats. Corresponding intestinal segments were collected from the SHAM group. Quantitative RT-PCR was performed using purified total RNA (n=5/group; animals were selected based on the availability of high-quality RNA from all tissue samples). Missing data were not quantified due to very low expression levels.
