# Supplementary material for: Effect of Roux-en-Y Gastric Bypass Surgery on Bile Acid Metabolism in Normal and Obese Diabetic Rats
Source: PLoS One. 2015 Mar 23;10(3):e0122273. doi: 10.1371/journal.pone.0122273 (PMC4370587; doi:10.1371/journal.pone.0122273)
Supplement: S1 Table — (PDF) [file pone.0122273.s006.pdf]

S2_Table Plasma and fecal bile acid profile on POD28 in SD rats

	Plasma (ng/mL), day 28			Fecal (μg), day 28		
	**SHAM**	**RYGB**	***P value***	**SHAM**	**RYGB**	***P value***
Unconjugated primary BA						
CDCA	57.6 (ND-100.7)	111.8 (38.8-360.8)	*0.123*	ND (ND-1.8)	4.5 (ND-16.2)	*0.017*
αMCA	87.8 (ND-171.6)	183.5 (43.7-702.7)	*0.233*	2.1 (ND-7.4)	ND (ND-8.1)	*0.596*
βMCA	67.4 (ND-129.1)	122 (75.7-516.9)	*0.107*	49.5 (13.2-91.5)	82 (13.3-91.4)	*0.578*
CA	332.4 (5.2-756.8)	628.4 (149.3-1324.3)	*0.233*	6.9 (4.2-9)	4.8 (2-8.8)	*0.596*
Conjugated primary BA						
T-CDCA	ND (ND-21.6)	3.4 (ND-28.9)	*0.107*	3 (0.6-12.2)	6.6 (2.9-11.2)	*0.6*
G-CDCA	ND (ND-27.8)	24.4 (ND-34.6)	*0107*	ND (ND-1)	1.1 (0.8-3.5)	*0.017*
T-αMCA	6.5 (ND-78.4)	31.6 (5.3-75.7)	*0.306*	ND (ND-0.6)	ND	*0.688*
T-CA	23.2 (7.4-259.5)	32.5 (11.4-306.1)	*0.627*	5.4 (2.3-15.3)	6 (0.3-7.2)	*1*
G-CA	ND	ND	*NA*	ND	ND	*NA*
Unconjugated secondary BA						
LCA	ND (ND-1.9)	1.3 (ND-5.3)	*0.127*	17.5 (7.7-75.2)	34.4 (3.1-70)	*0.901*
DCA	28 (ND-76.4)	80.7 (27-141.9)	*0.107*	50.4 (18.9-116.2)	43.6 (ND-108.5)	*0.942*
UDCA	16.3 (ND-41.8)	34.5 (23.8-166.9)	*0.086*	ND	ND	*NA*
Conjugated secondary BA						
T-LCA	ND	ND	*NA*	ND (ND-0.2)	ND	*1*
G-LCA	ND	ND	*NA*	0.097 (ND-1)	0.44 (0.02-1.6)	*0.401*
T-DCA	ND (ND-8.4)	ND (ND-13.6)	*0.451*	ND (ND-0.32)	0.15 (ND-0.83)	*0.271*
G-DCA	ND (ND-44.7)	39.1 (ND-143.9)	*0.107*	ND (ND-0.21)	0.027 (ND-0.11)	*0.272*
T-UDCA	ND	ND	*NA*	ND	ND	*NA*
G-UDCA	70.9 (12-545.3)	17.8 (17.3-42.4)	*0.233*	ND	ND	*NA*
CA/CDCA derived ratio	1.86 (0.92-3.09)	1.16 (0.80-1.31)	*0.163*	0.65 (0.43-2.57)	0.52 (0.21-0.71)	*0.271*

Data presented as Median (range); ND: not detected
